# Roles of IκB kinases and TANK-binding kinase 1 in hepatic lipid metabolism and nonalcoholic fatty liver disease

**DOI:** 10.1038/s12276-021-00712-w

**Published:** 2021-11-30

**Authors:** Jin Young Huh, Alan R. Saltiel

**Affiliations:** 1 Center for Adipose Tissue Remodeling, Institute of Molecular Biology and Genetics, Department of Biological Sciences, Seoul National University, Seoul, South Korea; 2 Department of Medicine, University of California, San Diego, CA 92093 USA; 3 Department of Pharmacology, University of California, San Diego, CA 92093 USA

**Keywords:** Obesity, Chronic inflammation

## Abstract

Nonalcoholic fatty liver disease (NAFLD) is the most common cause of chronic liver disease and is strongly associated with obesity-related ectopic fat accumulation in the liver. Hepatic lipid accumulation encompasses a histological spectrum ranging from simple steatosis to nonalcoholic steatohepatitis (NASH), which can progress to cirrhosis and hepatocellular carcinoma. Given that dysregulated hepatic lipid metabolism may be an onset factor in NAFLD, understanding how hepatic lipid metabolism is modulated in healthy subjects and which steps are dysregulated in NAFLD subjects is crucial to identify effective therapeutic targets. Additionally, hepatic inflammation is involved in chronic hepatocyte damage during NAFLD progression. As a key immune signaling hub that mediates NF-κB activation, the IκB kinase (IKK) complex, including IKKα, IKKβ, and IKKγ (NEMO), has been studied as a crucial regulator of the hepatic inflammatory response and hepatocyte survival. Notably, TANK-binding kinase 1 (TBK1), an IKK-related kinase, has recently been revealed as a potential link between hepatic inflammation and energy metabolism. Here, we review (1) the biochemical steps of hepatic lipid metabolism; (2) dysregulated lipid metabolism in obesity and NAFLD; and (3) the roles of IKKs and TBK1 in obesity and NAFLD.

## Obesity: tracing the inflammation underlying liver disease

A deeper understanding of the molecular processes underlying dysfunctional fatty-acid metabolism in nonalcoholic fatty liver disease (NAFLD) could help protect patients from severe liver damage. NAFLD is a common consequence of obesity, and severe disease can ultimately give rise to liver cancer or cirrhosis. Alan Saltiel of the University of California, San Diego, USA, and Jin Young Huh of Seoul National University, South Korea, have reviewed current knowledge of the mechanisms governing fatty acid metabolism in the liver, and how they are disturbed in obesity and NAFLD. The various IκB kinase (IKK) complexes and a protein known as TANK-binding kinase 1 (TBK1) play particularly prominent roles in the inflammatory response associated with NAFLD pathogenesis. Better insights into IKK- and TBK1-associated regulatory pathways could inform the development of new interventions for this currently untreatable condition.

## Introduction

The prevalence of obesity (excess fat storage) due to high-calorie food intake and a sedentary lifestyle is greatly increasing. Obesity is closely related to the prevalence of metabolic disorders, such as type 2 diabetes, cardiovascular disease, dyslipidemia, and nonalcoholic fatty liver disease (NAFLD)^1,2^. As obesity progresses, excess fat builds up in organs other than adipose tissue, and chronic hepatic fat accumulation induces fatty liver, which is currently the most common cause of obesity-related NAFLD^3,4^.

NAFLD is typically classified into nonalcoholic fatty liver (NAFL, simple steatosis) and nonalcoholic steatohepatitis (NASH)^5^. Hepatic steatosis is defined as an increase in hepatic fat that exceeds 5% of the liver’s weight seen on imaging or histology in the absence of alcohol intake, long-term steatogenic medication intake, or certain genetic diseases^5^. NASH is characterized by steatosis with hepatocyte injury in the form of ballooning hepatocytes, which is associated with hepatocyte death, apoptosis, and inflammation^5^. NASH patients can develop advanced liver disease, leading to cirrhosis and hepatocellular carcinoma (HCC)^6^. The global prevalence of NAFLD is over 25%, with the highest prevalence in the Middle East and South America and the lowest in Africa^3^. In the United States, the overall NAFLD prevalence in the adult population is projected to be 33% in 2030^7^. As there is still no efficient therapy for NAFLD, liver transplantation is currently the only treatment for advanced cirrhosis with liver dysfunction, which highlights the importance of understanding NAFLD development to find effective preventive and therapeutic strategies.

In this review, we summarize the molecular mechanism of hepatic fatty acid (FA) metabolism regulation under normal and NAFLD pathophysiological conditions. As hepatic inflammatory signaling is also a well-known factor regulating NAFLD progression, the role of the IκB kinase (IKK) complex, which is an inflammatory signaling hub, will be reviewed. In particular, the role of the IKK-related kinase TANK-binding kinase 1 (TBK1) in the regulation of lipid metabolism will be discussed.

## Hepatic FA metabolism

**Fig. 1:**
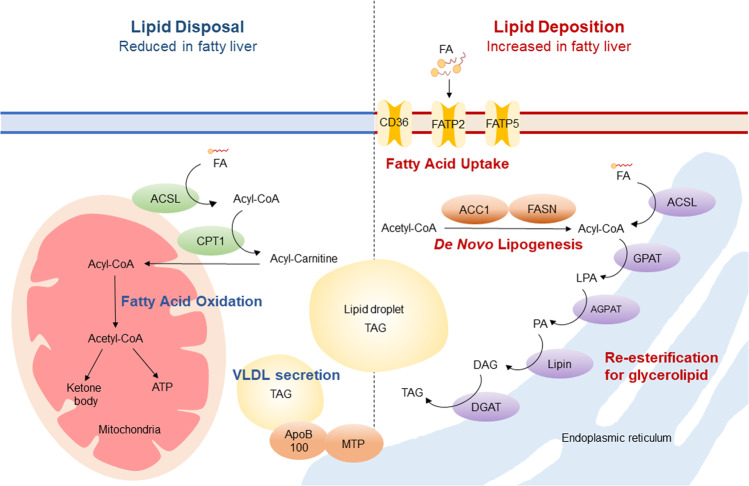
Hepatic lipid metabolism in fatty liver. Circulating FAs are imported via FATPs, including CD36, FATP2, and FATP5. ACSL converts the FAs taken up by cells to acyl-CoA, which is further reesterified to produce glycerolipids, mainly in the ER. In addition, FAs can be synthesized via DNL using acetyl-CoA. These intracellular FAs can be consumed to generate VLDL by forming a complex with ApoB100 via MTP. Furthermore, FAs are cleaved to generate acetyl-CoA through FA oxidation in the mitochondria to produce ATP and ketone bodies. An imbalance between lipid disposal and storage promotes fatty liver disease.

Hepatic steatosis results from an imbalance in lipid metabolism in hepatocytes, which is a major parenchymal cell type in the liver. Hepatocytes can maintain a steady lipid content of <5% of inflowing dietary or endogenous FAs^8^. This is attributable to the fact that FA uptake from the blood and de novo lipogenesis in the liver are counterbalanced by FA oxidation and release into the plasma as triglyceride (TG)-enriched very-low-density lipoprotein (VLDL)^9^. However, a preference for FA acquisition over lipid consumption via FA oxidation or VLDL secretion can lead to an increase in TG accumulation in cytosolic lipid droplets, which can promote steatosis (Fig. 1)^9,10^. Nonetheless, the molecular mechanisms driving abnormal hepatic lipid accumulation in NAFLD subjects remain elusive. As the regulation of hepatic lipid homeostasis is complex, we will first glance at some of the key regulators of NAFLD progression.

### FA uptake

Long-chain FA uptake is largely dependent on plasma membrane-associated FA transporters, with a minor contribution of passive diffusion^11^. Plasma membrane FA-binding protein (FABPpm), caveolin-1, CD36, and FA transporter protein are known to be involved in FA uptake^12,13^. The roles of FABPpm and caveolin-1 in the progression of steatosis through FA uptake have not been fully elucidated. Liver-specific CD36 knockout (KO) mice showed less steatosis upon high-fat diet (HFD) feeding, indicating that hepatic CD36 is involved in FA uptake^14^. In addition, CD36 and PPARγ expression levels were similarly elevated in obese rats with hepatic steatosis^15,16^.

Among the six FATP isoforms, FATP2 and FATP5 are the major FATPs in hepatocytes^17^. While FATP4 is not highly expressed in normal hepatocytes, its expression is induced in steatosis^18^. FATP2 overexpression in HepG2 cells elevated their FA uptake^19^. FATP5-deficient mice showed reduced progression of steatosis^20^, indicating a crucial role of FA transporters in hepatic lipid accumulation. To date, it is known that many FA transporters are involved in governing hepatic lipid levels, however, further investigation, including of the FA species transported by each transporter and the regulators of FA transporter activity, is needed.

### Acyl-CoA synthesis

FA uptake activity is closely related to acyl-CoA synthetase (ACS) activity because ACS-mediated consumption of intracellular FAs provides the driving force for FA uptake^21,22^. Intracellular FAs, including FAs taken up from the extracellular space, are conjugated with CoA by ACS, which makes them more hydrophilic and thus traps them inside the cells. Therefore, this conjugation is an important step for further FA metabolism^23^. ACS can be classified into very-long-chain ACS, long-chain ACS (ACSL), medium-chain ACS (ACSM), and short-chain ACS (ACSS), which have different substrate preferences but show significant overlap in chain-length specificity^23,24^. ACSL mainly catalyzes acyl-CoA synthesis with chain lengths of 12–20 carbons and comprises five isoforms (ACSL1, ACSL3, ACSL4, ACSL5, and ACSL6)^23,25^. While hepatocytes mainly express ACSL1, ACSL4, and ACSL5, ACSL1 has been suggested to contribute ~50% of hepatic ACSL activity^26,27^. ACSL1 has a substrate preference for palmitic acid and oleic acid, which are FA metabolites enriched in circulation^25^. Notably, liver-specific ACSL1 KO mice did not show significant changes in hepatic lipid levels, which may be due to diminished levels of FA re-esterification into triacylglycerol and FA oxidation^27^. ACSL1 in the liver can localize to both the endoplasmic reticulum (ER) and mitochondria, and various interacting proteins have been identified through mitochondrial or ER-targeted ACSL1 overexpression^28,29^. Based on the differential enrichment of FA oxidation-related proteins in the mitochondria and re-esterification enzymes in the ER, it has been suggested that the localization of ACSL1 can determine the metabolic fate of fatty acyl-CoA toward oxidation or re-esterification for lipid synthesis^28,29^; acyl-CoA produced by mitochondrial ACSL1 is oxidized, whereas acyl-CoA produced by ER ACSL1 is mostly used for re-esterification to diacylglycerol (DG) or TG. Consistent with this suggestion, adenovirus-mediated ACSL1 overexpression in rat primary hepatocytes increased FA reacylation and channeled FA toward DG and phospholipid synthesis, with elevated levels of ER-localized ACSL1^30^. Furthermore, ACSL1 directly interacts with carnitine palmitoyltransferase 1A in the rat liver^31^, which supports the crucial role of mitochondria-localized ACSL1 in FA oxidation. Nonetheless, the molecular mechanism by which ACSL1 localization in hepatocytes is regulated remains to be investigated.

### Re-esterification for lipid synthesis

Activated acyl-CoA can be reesterified for lipid synthesis^32^. The enzymes catalyzing re-esterification, including glycerol-3-phosphate acyltransferase (GPAT), 1-acylglycerol-3-phosphate acyltransferase (AGPAT), phosphatidic acid (PA) phosphohydrolase (PAPases/lipins), and diacylglycerol acyltransferase (DGAT), are mainly localized in microsomes and the ER^29^. The key rate-limiting step for re-esterification is lysophosphatidic acid (LPA) synthesis by mitochondrial and microsomal GPAT^22,33^. LPA can be converted to PA through AGPAT activity. PA can then be used as a precursor of phospholipids, such as phosphatidylethanolamine or phosphatidylcholine^9,29^. In addition, PA can be converted to DG via lipins, which have PAPase activity. DGAT activity esterifies additional FAs into DG to synthesize TG. Reesterified FAs can subsequently be transported to the cytosol and stored as cytosolic lipid droplets or form and secrete VLDL complexed with apoproteins into the circulation^9,29^.

Loss- and gain-of-function studies of these key enzymes have shown the crucial regulatory role of re-esterification in NAFLD progression. GPAT is responsible for the first step in re-esterification. There are four isoforms, GPAT1, GPAT2, GPAT3, and GPAT4, which differ in subcellular localization and substrate preferences^34^. GPAT1 and GPAT2 are localized in the mitochondria and account for 30–50% of total hepatic GPAT activity^34^. GPAT1 expression is elevated in response to insulin but is reduced upon fasting-related cAMP-dependent signaling^35,36^. GPAT1-deficient mice showed increased hepatic FA oxidation with enhanced circulating levels of ketone bodies^37^. Concomitantly, hepatic TG levels declined by 60%, which revealed the pivotal role of GPAT1 in the management of hepatic TG synthesis. In contrast, GPAT3 and GPAT4 are involved in microsomal GPAT activity in the liver^38^. Although GPAT4-deficient mice show reduced hepatic TG levels^39^, the roles of GPAT3 and GPAT4 in the regulation of hepatic lipid metabolism and their roles in NAFLD progression remain to be further elucidated.

DGAT1 is primarily involved in TG synthesis in conjunction with microsomal luminal activity for VLDL formation, whereas DGAT2 utilizes cytosol-accessible activity to store TG in cytosolic lipid droplets^40^. Intracellular TG levels increased when DGAT1 was overexpressed in rat hepatoma cells^41^, and HFD-induced steatosis was reduced in DGAT1 KO mice^42^. Furthermore, liver-specific DGAT2 KO mice showed suppressed steatosis upon HFD feeding^43^. Accordingly, DGAT2 inhibitor administration reduced hepatic steatosis in healthy adults^44^. These studies suggest that increased hepatic DGAT activity may be a crucial factor in promoting steatosis.

### De novo lipogenesis

In both NAFLD patients and the HFD-fed mouse model, newly synthesized FAs resulting from DNL have been reported to increase^45,46^. DNL is initiated by ATP-citrate lyase (ACLY), which converts citrate to acetyl-CoA^47^. Acetyl-CoA carboxylase (ACC) synthesizes malonyl-CoA, which is a substrate for FA synthase (FASN) to generate palmitic acid. ACC has two isoforms, ACC1 in the cytoplasm and ACC2 in the mitochondria. ACC1 facilitates the synthesis of cytosolic malonyl-CoA, which is used as a substrate for FASN^47^. ACC2 mediates malonyl-CoA synthesis in the mitochondria, which inhibits carnitine palmitoyltransferase 1 (CPT1), the key enzyme of FA oxidation^48^. While ACC1 whole-body KO mice are embryonic lethal^49^, liver-specific ACC1 KO mice showed 70% reduced malonyl-CoA levels and significantly decreased hepatic TG levels^50^. FASN produces palmitate by affixing malonyl-CoA to acetyl-CoA in a series of condensation events, which are key rate-limiting steps in DNL^51^. FASN whole-body KO mice are embryonic lethal^52^, but liver-specific FASN KO mice showed attenuated hepatic DNL activity, resulting in the deposition of hepatic malonyl-CoA^53^. Interestingly, liver-specific FASN KO mice fed a zero-fat diet to stimulate DNL showed exacerbated hypoglycemia and hepatic steatosis, which were rescued upon administration of a PPARα agonist (WY-14.643)^53^. These studies indicate that newly synthesized FAs mediated by FASN act as PPARα ligands to regulate hepatic FA oxidation and gluconeogenesis. In NAFLD patients, 10-day administration of TVD-2640, a FASN inhibitor, resulted in significant reductions in ALT levels and DNL^54^. Therefore, the functional modulation of regulatory enzymes mediating the DNL pathway, such as ACC and FASN, has an important role in steatosis development.

Hepatic DNL is primarily controlled by transcriptional regulation^55^. ACC and FASN expression levels are synergistically controlled by carbohydrate-responsive element-binding protein (ChREBP) and sterol regulatory element-binding protein (SREBP-1c)^56^. SREBP-1c, a key transcriptional modulator of lipogenic enzymes, is activated by insulin in response to feeding, thereby increasing the expression of DNL-related genes^57^. SREBP-1c expression is regulated by the liver X receptor (LXR)^58^. ChREBP expression is strongly reduced under fasting conditions, whereas it is activated by a high-glucose or high-carbohydrate diet, increasing the expression of DNL-related genes^59^. ChREBP whole-body KO mice showed reduced mRNA levels of hepatic lipogenic enzymes, such as ACLY, ACC1, and FASN^60^. Furthermore, the expression of lipogenic genes, such as LXRα, SREBP-1c, ACC1, and FASN, is increased in the liver tissues of NAFLD patients^61^.

### VLDL secretion

Hepatic TG can be derived from circulating lipids, such as albumin-bound FAs, VLDL remnants, chylomicrons, and DNL in the liver^62^. Hepatic TG is released into the bloodstream primarily in the form of VLDL, which contains both lipids and apoproteins, including apoB100^63^. Secreted VLDLs provide an energy source to other tissues, such as muscle and adipose tissues^64^. VLDL is formed in two steps. The first step is lipidation of apoB100 catalyzed by microsomal TG transfer protein (MTP), which is processed in the ER. The nascent VLDL particles are subsequently transported to the Golgi apparatus, where they are further lipidated until they grow into mature VLDL particles^65^. Thus, apoB100 and MTP are vital for hepatic VLDL secretion and lipid homeostasis. In obese subjects, altered hepatic TG levels affect plasma VLDL levels by augmenting the amount of substrate, enhancing MTP activity, or influencing apolipoprotein B synthesis or breakdown^62,66^. In nondiabetic obese NAFLD patients, VLDL secretion is doubled compared to that in normal subjects^67^. However, even with a substantial amount of hepatic lipids, steatosis can worsen if secretion is hampered. For example, whereas *ob/ob* animals show steatosis, their VLDL secretion is lowered^68^. Thus, maintaining a balance between hepatic TG and VLDL secretion can be an important aspect of managing hepatic lipid levels.

### FA oxidation

Hepatic FA oxidation occurs in the mitochondria, peroxisomes, and ER, depending on the FA species. Very long-chain (C20–C26) and branched-chain FAs are mainly oxidized in peroxisomes, whereas β-oxidation of long-chain FAs primarily occurs in mitochondria^9^. Long-chain fatty acyl-CoA, synthesized by ACS using ATP and CoA, can be oxidized in the mitochondria to be cleaved into acetyl-CoA. Acetyl-CoA then enters the tricarboxylic acid (TCA) cycle to produce NADH and FADH_2_^23^, which serve as substrates for electron transfer chains in the mitochondrial inner membrane, exploiting proton gradients in the intermembrane space to generate ATP. As acyl-CoA requires a carnitine shuttle to enter the mitochondria, it is converted to palmitoyl carnitine by CPT1 at the mitochondrial outer membrane^69^ and then enters the mitochondrial inner membrane matrix via carnitine-acylcarnitine translocase (CAT) and is converted back to acyl-CoA by CPT2^29^. Acyl-CoA then undergoes β-oxidation through four enzymatic reactions catalyzed by acyl-CoA dehydrogenase, 2-enoyl-CoA hydratase, 3-hydroxyacyl-CoA dehydrogenase, and 3-ketoacyl-CoA thiolase^70^.

In response to starvation, FA oxidation and ketogenesis are actively induced in the liver^69^. The substrate concentration and/or affinity of an enzyme for its substrates, such as carnitine and long-chain acyl-CoAs, affect the activity of mitochondrial FA oxidation^23,71^. In particular, CPT1 is a key rate-limiting enzyme involved in mitochondrial FA oxidation^72^. CPT1 is regulated at multiple levels, with PPARα promoting *Cpt1* mRNA expression during fasting, which leads to enhanced CPT1 enzyme activity^73–75^. In addition, alterations in tissue malonyl-CoA levels and in enzyme susceptibility to malonyl-CoA inhibition modulate hepatic CPT1 activity^76,77^. This feedback mechanism can effectively govern the fate of FAs toward oxidation or synthesis, considering that malonyl-CoA is an intermediate metabolite of DNL.

## Obesity-associated adipose tissue dysfunction and NAFLD

**Fig. 2:**
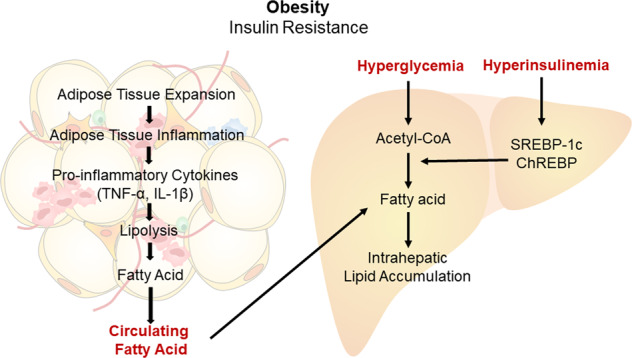
Obesity-induced adipose tissue inflammation and NAFLD. Excess energy intake promotes adipose tissue expansion, which is closely associated with elevated pro-inflammatory responses in adipose tissue through the recruitment of various immune cells. In turn, these cells enhance the levels of proinflammatory cytokines, such as TNF-α and IL-1β. Inflammatory signaling stimulates adipocyte lipolysis, thereby increasing FA influx into the liver. Moreover, insulin resistance in obese subjects mediates hyperglycemia and hyperinsulinemia, which stimulates DNL by supplying substrates and inducing the activation of key lipogenic transcription factors (SREBP-1c and ChREBP). All of these factors (increased plasma FA levels, hyperglycemia, and hyperinsulinemia) exacerbate lipid accumulation in the liver.

In NAFLD patients, the majority (59%) of hepatic fat is derived from nonesterified FAs (NEFAs), 26% from DNL, and 15% from diet^78^. In particular, plasma FAs are the major source of hepatic TGs during fasting. Considering the increased blood NEFA level due to elevated lipolysis in the adipose tissue of obese subjects^79^, proper modulation of circulating NEFA levels may be used to avoid NAFLD development. The obesity-induced inflammatory response in adipose tissue is well known to contribute to enhanced basal lipolysis^80^. During excessive energy accumulation in obese adipose tissue, inflammatory factor expression is induced by hypoxia, ER stress, and enlarged adipocytes^79,80^. This not only stimulates proinflammatory responses in adipose tissue macrophages but also leads to increased infiltration of circulating monocytes into obese adipose tissue^81–83^. In addition to macrophages, immune cells (such as neutrophils, Th1 T cells, and CD8 T cells) increase, while anti-inflammatory immune cells (such as M2 macrophages, regulatory T cells, and invariant natural killer T cells) decrease, ultimately exacerbating adipose tissue inflammation^79,84^. Moreover, proinflammatory cytokines, such as TNFα and IL-1β, are elevated in obese adipose tissue and stimulate various inflammatory signaling pathways, including JNK and NF-κB, accelerating insulin resistance^85^. This eventually suppresses the crucial function of adipocytes as an energy reservoir and leads to increased basal lipolysis, which mediates increased blood NEFA levels. This may also drive ectopic fat accumulation in other organs, such as the liver, muscle, and heart, and may lead to steatosis of NAFLD (Fig. 2).

Insulin resistance caused by obesity can result in hyperinsulinemia, which can enhance DNL via transcriptional activation of SREBP-1c or ChREBP^86,87^. Furthermore, increased blood glucose levels, which can be induced by inefficient insulin-mediated suppression of hepatic glucose production, contribute to the supply of substrates for DNL^88^. In addition to the rise in basal lipolysis caused by inflammation, the suppression of lipolysis by insulin is impeded in the setting of insulin resistance^80^, resulting in increased circulating FA levels. Furthermore, circulating FAs can increase hepatic acetyl-CoA levels through enhanced FA uptake into the liver. Inefficient hepatic lipid disposal eventually leads to the accumulation of acetyl-CoA, promoting DNL and conversion to FA metabolites, including DG, which may suppress insulin receptor signaling through PKCε activation (Fig. 2)^11,89^. Therefore, deteriorating hepatic steatosis resulting from enhanced lipid anabolism and reduced lipid catabolism, which can be accelerated by various stress responses and insulin resistance, can progress to NAFLD.

### Liver inflammation and IκB kinases

Short-term inflammation induced by an acute stimulus in the liver can have beneficial effects on hepatocyte survival and wound healing; however, chronic hepatic inflammation accelerates hepatocyte dysfunction and fibrosis by inducing hepatocyte injury and inflammatory responses^90^. Of note, NF-κB is one of the primary regulators of liver inflammation, and its activity is upregulated, which is an important factor leading to NAFLD^91^.

**Fig. 3:**
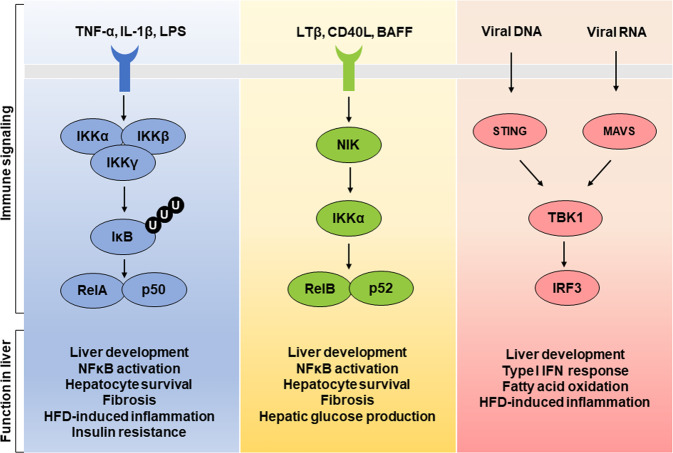
IKK and TBK1 functions in the liver. Canonical NF-κB signaling is mediated by the IKKα/IKKβ/IKKγ complex upon proinflammatory cytokine (TNF-α or IL-1β) or lipopolysaccharide stimulus. This complex induces the phosphorylation-mediated degradation of IκB, which leads to NF-κB activation. Furthermore, LTβ, CD40L, and BAFF stimulate NIK phosphorylation, thereby promoting IKKα phosphorylation and downstream signaling via the RelB:p52 complex. On the other hand, TBK1 is phosphorylated via STING or MAVS activation upon viral infection, which promotes IRF3 activation for the type I IFN response. Additionally, inactive TBK1 promotes FA oxidation in hepatocytes.

There are five NF-κB family members: NF-κB1 (p105 and p50), NF-κB2 (p100 and p52), c-Rel, RelA, and RelB. Each has a Rel homology domain, which is involved in DNA binding, dimerization, and interactions with IκB^92^. NF-κB signaling can be classified into canonical and noncanonical pathways. Canonical pathways are activated by stimuli such as lipopolysaccharides or inflammatory cytokines, including TNF-α and IL-1β. Initiation of the canonical pathway via Toll-like receptor or cytokine receptor signaling depends on the IKK complex formed by IKKα, IKKβ, and IKKγ (NEMO) to mediate IκB phosphorylation-mediated degradation, which liberates NF-κB and stimulates nuclear localization to induce target gene expression (inflammatory cytokines, growth factors, and cell adhesion molecules) (Fig. 3)^93^. Noncanonical NF-κB signaling is driven by ligands such as BAFF, CD40L, LTα1β2/LIGHT, and RANKL^94^. Ligand stimulation activates an IKKα homodimer via NF-κB-inducing kinase (NIK), which cleaves p100 to p52, triggering transcription via the p52:RelB complex. Noncanonical NF-κB signaling plays an important role in the development of B cells and lymphoid organs (spleen, lymph nodes, mucosal lymphoid tissue)^94^. Given NF-κB signaling is closely associated with hepatic inflammation, the roles of IKKs in hepatocyte survival, inflammation, and energy metabolism have been investigated using diverse genetic animal models.

#### Hepatic IKKβ

IKK is the major signaling factor mediating the inflammatory response, and it could impact NAFLD progression. Whole-body IKKβ KO mice are embryonic lethal due to massive liver apoptosis^95,96^. IKKβ deficiency diminishes not only basal NF-κB activation but also TNFα-induced activation, which suggests a key role for IKKβ in liver development and NF-κB signaling. Liver-specific IKKβ KO mice exhibited normal liver function with little residual NF-κB activity^97^. However, hepatic apoptosis was elevated in liver-specific IKKβ KO mice upon concanavalin A administration, increasing circulating TNFα levels. Furthermore, in a DEN (diethylnitrosamine)-induced HCC model, liver-specific IKKβ KO increased hepatocyte death, demonstrating that IKKβ has antiapoptotic activity in the liver^98^. On the other hand, HFD-fed liver-specific IKKβ KO mice showed improved glucose intolerance and insulin-mediated hepatic glucose production suppression compared to control mice^99^. Concomitantly, HFD-induced inflammatory gene expression was reduced in the KO mice. Also, transgenic mice with liver-specific constitutively active IKKβ developed insulin resistance and showed elevated secretion of hepatic cytokines, such as IL-1 and TNF-α^91^. These data suggest that hepatic IKKβ mediates HFD-induced insulin resistance and inflammation. Hepatic IKKβ has also been shown to promote intracellular lipidation by activating ChREBP, which can lead to enhanced VLDL secretion and, consequently, higher plasma TG levels^100,101^. Furthermore, liver-specific constitutively active IKKβ transgenic mice demonstrated significant increases in NF-κB signaling, inflammatory cell recruitment, and fibrosis^102^. These findings indicate that hepatic IKKβ inhibition may be a therapeutic target for NAFLD treatment. For example, in high sucrose diet-induced NASH model mice, administration of a pharmacological IKKβ inhibitor (AS602868) reduced weight gain, adipose tissue weight, and inflammatory cytokine production^103^.

#### Hepatic NEMO

NEMO binds to IKKα and IKKβ as a noncatalytic scaffolding subunit to mediate IκB phosphorylation, which has an essential role in canonical NF-κB signaling^104,105^. Like IKKβ KO mice, whole-body NEMO KO mice are embryonic lethal due to severe apoptosis-mediated liver damage, along with compromised IκB phosphorylation and NF-κB activity^106^. Complete NF-κB inactivation with increased hepatocyte death in hepatocyte-specific NEMO-deficient mice demonstrated that NEMO-mediated NF-κB signaling is essential for hepatocyte survival^107^. Of note, liver-specific NEMO KO mice gained less body weight but had exacerbated hepatic macrovascular steatosis with elevated apoptosis and proinflammatory cytokine expression^108^. These mice also showed increased spontaneous tumorigenesis, which reveals the important interaction between hepatocyte lipid metabolism and inflammation and their contribution to the regulation of tumorigenesis. However, the detailed mechanisms underlying these interactions have not yet been elucidated.

#### Hepatic NIK

NIK has a key role in noncanonical NF-κB signaling via IKKα phosphorylation. IKKα phosphorylation is increased in liver tissues from leptin-deficient *ob*/*ob* mice^109^. Whole-body NIK KO mice show lowered blood glucose levels and reduced pyruvate tolerance, which may indicate suppressed gluconeogenesis^109^. Mechanistically, hepatic NIK enhances the phosphorylation-mediated stability of CREB, which is one of the key transcription factors regulating fasting-induced gluconeogenic genes. In the context of increased IKKα activity in obese liver tissue, IKKα is involved in increasing blood glucose levels by stimulating hepatic glucose production. On the other hand, hepatocyte-specific NIK-overexpressing transgenic mice exhibit massive liver inflammation, oxidative stress, hepatocyte apoptosis, and liver fibrosis^110^. Overexpression of a mutant NIK (G885R) lacking catalytic activity for noncanonical NF-κB activation promoted hepatocyte apoptosis to the same extent as wild-type NIK overexpression, suggesting noncanonical NF-κB-independent regulation by NIK. Liu et al. assessed the roles of NIK in different cell types using hepatocyte-specific KO and myeloid cell-specific KO mice^111^. Both NIK-deficient mouse models showed comparable levels of hepatic steatosis and inflammation. Interestingly, Mx1-cre driver-mediated NIK deficiency in both hepatocytes and immune cells diminished hepatic inflammation and steatosis with reduced expression of lipogenic genes, such as FASN, ACC1, and SREBP-1c^111^. These data reveal that NIK mediates the interaction between hepatocytes and immune cells in the modulation of steatosis. However, further studies are needed to determine whether NIK exerts this function via the noncanonical NF-κB response and to unravel the mechanism controlling hepatic inflammation.

### IKK-related kinase TBK1

TBK1 is an IKK-related kinase along with IKKε, and they exhibit 61% sequence identity^112^. TBK1, also known as NAK or T2K, is structurally similar to IKKβ. However, as TBK1 lacks the NEMO-binding domain that allows IKKβ to bind NEMO to form the IKK complex for IκB phosphorylation, it has been speculated that TBK1 has a distinct substrate preference^112,113^. TBK1 is composed of an N-terminal kinase domain (KD), a ubiquitin-like domain (ULD), and a C-terminal scaffolding/dimerization domain (SDD), and this domain arrangement appears to be shared among IKK family members^93,114^. These three domains combine to form a tripartite architecture upon dimerization, allowing the active kinase sites to face away from one another^114,115^. Upon upstream signaling, TBK1 is recruited to the signaling complex for activation^116^. A high local concentration of TBK1 allows interdimer KD interactions that lead to trans-autophosphorylation^114,115^. Once activated, phosphorylated TBK1 can rapidly phosphorylate the remaining TBK1 pool to form fully activated kinase dimers^115^. Both adaptor protein recruitment upon upstream signaling and the KD back-to-back structure of the TBK1 dimer provides fine-tuning regulatory mechanisms for TBK1 activity.

TBK1 substrate specificity appears to be regulated within the KD because removal of the ULD and SDD does not alter the specificity of TBK1^114^. In addition, subcellular localization may be a key determinant of TBK1 substrate specificity^115,116^. As the adaptors have unique binding partners, they recruit TBK1 to distinct signaling complexes and cellular locations, thereby directing TBK1 activity toward specific downstream pathways^115^. Thus, TBK1 can modulate various downstream substrates, which support the diverse functions of TBK1 through the formation of several different complexes. TBK1 has critical roles in the regulation of immune responses, autophagy, and energy metabolism.

#### Innate immune responses

TBK1 and IKKε have been explored as mediators of innate immune responses that mediate antiviral responses through interferon regulatory factor 3 (IRF3) phosphorylation^117,118^. The two IKK-related kinases have similar enzymatic features and require Ser172 phosphorylation for kinase activity^119^. TBK1 and IKKε have a redundant function in the antiviral response, although they are expressed at different levels, depending on the cell type^120^. Upon viral or bacterial infection, cytoplasmic DNA is sensed by cyclic GMP-AMP synthase, which synthesizes cGAMP, which can oligomerize stimulator of interferon genes (STING), which recruits and activates TBK1. Activated TBK1 phosphorylates STING, which leads to the phosphorylation of IRF3 and IRF7 to induce the expression of type I interferons and other cytokines (Fig. 3)^121^. In addition, TBK1 mediates antiviral responses by interacting with mitochondrial antiviral signaling protein (MAVS) in mitochondria^122^. Thus, downstream TBK1 signaling may be affected by its localization.

#### Autophagy

Autophagy is a self-digestion mechanism responsible for the removal of damaged organelles, malformed proteins, and nonfunctional long-lived proteins via lysosomal degradation of autophagosomes^123^. TBK1 is known as a key regulator of autophagy. In the context of innate immune response regulation, TBK1 has been shown to promote lysosomal degradation of ubiquitinated bacteria via interaction with NDP52 or optineurin (OPTN)^124–126^. In addition, phosphorylation of p62, known as an autophagic cargo adaptor protein, at Ser403 is critical for autophagic maturation, and TBK1 has been demonstrated to induce p62 Ser403 phosphorylation^127,128^.

Furthermore, impaired lipophagy, which is a selective degradation of intracellular lipid droplets by lysosomes, has been suggested to be linked to NAFLD progression^129–131^. Recently, it has been reported that increased SREBP-1c expression in response to an HFD suppresses cystathionine gamma-lyase-mediated ULK1 sulfhydration, which can exacerbate hepatic steatosis via reduced lipophagy^132^. However, whether TBK1 can modulate lipophagy in hepatocytes has not yet been elucidated.

Mitophagy is a type of selective autophagic regulation that removes depolarized or damaged mitochondria. If mitophagy is not properly coordinated, it can augment damaged mitochondrial accumulation, cellular dysfunction, mitochondria-mediated ATP deprivation, and oxidative stress responses^133–135^. TBK1 binds to polyubiquitinated mitochondria and promotes autophagosome engulfment via p62 Ser403 phosphorylation to mediate PINK/PARKIN-dependent mitophagy^136^. TBK1 autophosphorylation upon mitophagy induction is reported to occur first, followed by the recruitment of adaptor proteins, such as OPTN, NDP52, and p62, suggesting that TBK1 has key regulatory functions in the early phases of mitophagy^137,138^. PINK1/PARKIN-dependent and PINK1/PARKIN-independent pathways have been investigated as mitophagy regulatory mechanisms^139^. In C2C12 skeletal muscle cells, treatment with a mitochondrial depolarization reagent (carbonyl cyanide m-chlorophenyl hydrazone) induced AMPK activation along with TBK1 activation via the PINK1/PARKIN-independent pathway, which led to autophagosomal engulfment^140^. Although mitochondrial numbers have been recently found to be increased in liver-specific TBK1 KO mice, more research is needed to evaluate whether the alterations are driven by impaired mitophagy^141^.

#### Energy metabolism

TBK1 has a ubiquitous tissue distribution, while IKKε is more strongly expressed in immune cells^120,142^. IKKε is involved in obesity-related energy metabolism. IKKε expression has been shown to be higher in the adipose tissue and liver of obese mice, and whole-body IKKε KO mice showed reduced HFD-induced body weight gain and adipose tissue expansion and thus lower adipose inflammatory responses^143^. In addition, increased activities of IKKε and TBK1 can lead to obesity-mediated catecholamine resistance, primarily in adipocytes, by mediating PDE3B phosphorylation to restrict cAMP levels^142^. However, the specific role of hepatic IKKε in the regulation of lipid metabolism needs to be further investigated.

To clarify the function of TBK1 in the regulation of energy metabolism and obesity-induced inflammation in adipocytes and hepatocytes in vivo, adipocyte- and hepatocyte-specific TBK1 KO mice have been studied. Adipocyte-specific TBK1 KO mice gained less body weight due to increased energy expenditure but showed elevated adipose tissue inflammation with increased insulin resistance upon HFD feeding^144^. Mechanistically, adipocyte TBK1 mediates the inhibitory phosphorylation of AMPK and phosphorylates NIK, which results in increased energy expenditure with enhanced adipose inflammation in these mice.

In contrast, hepatic TG accumulation is significantly increased in liver-specific TBK1 KO (LTKO) mice upon both a normal chow diet and HFD feeding, with comparable body weights^141^. In particular, after a 16-h fast, hepatic TG accumulation in LTKO mice was increased due to impaired fasting-stimulated FA oxidation. Fasting-stimulated mitochondrial localization of ACSL1 was suppressed, thereby diminishing FA channeling for subsequent FA oxidation in LTKO mice. Conversely, ER-localized ACSL1 was enriched in the LTKO mouse liver, with enhanced re-esterification activity for TG synthesis. The suppression of FA oxidation with enhanced re-esterification activity in LTKO mice supports the important regulatory role of TBK1 in ACSL1 localization in the two organelles. Notably, fasting increased nonphosphorylated (inactive) TBK1 in hepatocytes. In addition, kinase-dead TBK1 (K38A) showed a higher binding affinity for ACSL1. Moreover, kinase-dead TBK1 more efficiently restored FA oxidation activity in TBK1-deficient primary hepatocytes than WT TBK1, supporting the role of inactive TBK1 in fasting-induced FA oxidation as a scaffolding protein for ACSL1. Although it has been suggested that inactive TBK1 interacts with ACSL1 with greater binding affinity, more research is needed to determine how mitochondrial localization is governed by TBK1. Furthermore, hepatic lipid accumulation has been observed in LTKO mice without inflammatory gene activation during NCD feeding; however, the increase in inflammation-related marker gene expression in HFD-fed LTKO mice may indicate that hepatic steatosis can precede inflammation in NAFLD progression.

## Conclusion

Obesity-related inflammation and insulin resistance are associated with hepatic lipid accumulation and systemic inflammation, which contribute to NAFLD development. NAFLD occurs when FA oxidation and lipid export fail to compensate for increased lipid uptake and synthesis. In addition to hepatic lipid accumulation, the inflammatory response is a crucial factor in NAFLD pathogenesis. In particular, IKKs and TBK1 play crucial roles in hepatic inflammation and lipid homeostasis, consequently modulating NAFLD progression. Nonetheless, the question of how inflammatory signaling affects lipid metabolism and vice versa is largely unanswered. Given that, emerging evidence supports the pivotal functions of TBK1 in governing the inflammatory response, energy metabolism, and autophagy, TBK1 might serve as a signal integration point in response to various environmental stimuli. Notably, considering the regulatory function of TBK1 in the autophagy process, its effects on lipid metabolism, including lipophagy, remain to be determined in future studies. In addition, to understand NAFLD pathogenesis, it is necessary to unravel the molecular mechanisms underlying the cooperative regulation of lipid metabolism and inflammation, thus identifying potential therapeutic targets for improving NAFLD progression.
